# Adaptive ultrasound temperature imaging for monitoring radiofrequency ablation

**DOI:** 10.1371/journal.pone.0182457

**Published:** 2017-08-24

**Authors:** Yi-Da Liu, Qiang Li, Zhuhuang Zhou, Yao-Wen Yeah, Chien-Cheng Chang, Chia-Yen Lee, Po-Hsiang Tsui

**Affiliations:** 1 School of Electronic Information Engineering, Tianjin University, Tianjin, China; 2 College of Life Science and Bioengineering, Beijing University of Technology, Beijing, China; 3 Faculty of Information Technology, Beijing University of Technology, Beijing, China; 4 Institute of Applied Mechanics, National Taiwan University, Taipei, Taiwan; 5 Department of Electrical Engineering, National United University, Miao-Li, Taiwan; 6 Department of Medical Imaging and Radiological Sciences, College of Medicine, Chang Gung University, Taoyuan, Taiwan; 7 Medical Imaging Research Center, Institute for Radiological Research, Chang Gung University and Chang Gung Memorial Hospital at Linkou, Taoyuan, Taiwan; 8 Department of Medical Imaging and Intervention, Chang Gung Memorial Hospital at Linkou, Taoyuan, Taiwan; Nanjing University, CHINA

## Abstract

Radiofrequency ablation (RFA) has been widely used as an alternative treatment modality for liver tumors. Monitoring the temperature distribution in the tissue during RFA is required to assess the thermal dosage. Ultrasound temperature imaging based on the detection of echo time shifts has received the most attention in the past decade. The coefficient *k*, connecting the temperature change and the echo time shift, is a medium-dependent parameter used to describe the confounding effects of changes in the speed of sound and thermal expansion as temperature increases. The current algorithm of temperature estimate based on echo time shift detection typically uses a constant *k*, resulting in estimation errors when ablation temperatures are higher than 50°C. This study proposes an adaptive-*k* algorithm that enables the automatic adjustment of the coefficient *k* during ultrasound temperature monitoring of RFA. To verify the proposed algorithm, RFA experiments on *in vitro* porcine liver samples (total *n* = 15) were performed using ablation powers of 10, 15, and 20 W. During RFA, a clinical ultrasound system equipped with a 7.5-MHz linear transducer was used to collect backscattered signals for ultrasound temperature imaging using the constant- and adaptive-*k* algorithms. Concurrently, an infrared imaging system and thermocouples were used to measure surface temperature distribution of the sample and internal ablation temperatures for comparisons with ultrasound estimates. Experimental results demonstrated that the proposed adaptive-*k* method improved the performance in visualizing the temperature distribution. In particular, the estimation errors were also reduced even when the temperature of the tissue is higher than 50°C. The proposed adaptive-*k* ultrasound temperature imaging strategy has potential to serve as a thermal dosage evaluation tool for monitoring high-temperature RFA.

## Introduction

Liver cancer is a common cause of cancer mortality [1]. Hepatocellular carcinoma (HCC) accounts for 85%–90% of primary liver cancers [2]. Surgical resection and liver transplants are currently considered mainstream HCC treatments; however, not every patient is suitable for undergoing these treatments due to clinical considerations. In such a situation, minimally invasive radiofrequency ablation (RFA) is used as the primary alternative modality for clinical HCC treatment [3–6]. In RFA, a radiofrequency (RF) electrode is inserted into the target tumor; subsequently, the electrode delivers a strong alternating current that produces heat and increases the temperature of the tissue, resulting in protein denaturation and coagulation necrosis [7–8].

During clinical RFA, the insertion of the RF electrode into the tumor is widely guided by computed tomography (CT) and ultrasound B-mode imaging. Compared with CT, ultrasound has the advantages of flexibility, relative availability, low cost, and real-time feedback capability [9–10]. Note that RFA-induced high temperature typically results in the formation of gas bubbles in the ablation zone. Gas bubbles contribute a significant acoustic impedance mismatch, providing additional ultrasound backscattered components to alter the speckle pattern and strengthen the brightness of ultrasound B-mode imaging. Thus, B-scan and some parametric imaging techniques can be used to observe these bubbles to evaluate the ablation zone and its size [11]. Thanks to the advances in data analysis methods, ultrasonography has gradually become popular and attractive in monitoring RFA.

Besides the information of gas bubbles, a precise assessment of the RFA outcome depends on the evaluation of the thermal dosage in the tissue. In practical applications, estimating temperature changes in the ablation zone is highly required. Physically, the temperature increase not only results in the thermal expansion of the tissue but also affects its scattering properties, thereby altering the acoustic parameters of ultrasound propagation, such as speed of sound (SOS) [12–15], acoustic attenuation [16–17], and the backscattered energy of reflected echoes [18–20]. In particular, as the temperature increases in tissues, the thermal expansion and changes in SOS result in echo time shift. In the past decade, using the echo time shift for ultrasound temperature imaging has received the most attention, and it has been successfully employed to visualize temperature variation during RFA under the assumption that the coefficient *k*—a parameter used to describe the effects of changes in the SOS and tissue thermal expansions—is a time-invariant constant [21–23]. Note that this assumption may not stand when the tissue temperature exceeds 50°C. It has been shown that the coefficient *k* remains nearly constant from 37°C to around 50°C [24]. A constant *k* represents that the dependency of SOS on temperature follows a linear relationship. However, at temperatures above about 50°C, the effect of thermal expansion should be considered, and the nonlinear dependence of the SOS on temperature makes uncertainty of temperature estimation much higher [25]. Considering that the ablation temperature of RFA used clinically is close to the boiling point, using a constant *k* to estimate the temperature during RFA may not be applicable and possibly result in bias in echo time shift estimation.

To improve the performance of echo time shift detection using the constant *k* in monitoring RFA, this study developed ultrasound temperature imaging algorithm based on an adaptive estimation of *k*. Here, the term “*adaptive*” indicates that the coefficient *k* can be adaptively adjusted during RFA in an automated manner. In the next section, we introduce the theory underlying the echo-shift ultrasound temperature estimation and then explain how we designed the adaptive-*k* algorithm. The temperatures of liver samples *in vitro* used for RFA with different powers were estimated using ultrasound temperature imaging based on the constant- and adaptive-*k* methods, and measured by infrared camera and thermocouples for comparisons. The results demonstrated that the adaptive-*k* echo-shift temperature imaging monitored RFA favorably, even when the temperature was higher than 50°C.

## Materials and methods

### Ultrasound temperature imaging using the adaptive k

Increasing the temperature in the tissue changes the SOS and thermal expansion, which results in an echo time shift (i.e., signal time delay) [12–13]. The relationship between the echo time shift and temperature can be described as [14]

$$\delta T(z)=\frac{c_{0}(z)}{2}\left(\frac{1}{\alpha (z)-\beta (z)}\right)\frac{\partial }{\partial z}\left(\delta t(z)\right),$$ (1)

Where *δT*(*z*) = *T*(*z*)–*T*_0_ represents the temperature change at depth *z*; *T*_0_ is the initial temperature; *δt*(*z*) represents the echo time shift; *α*(*z*) is the coefficient of the tissue’s thermal expansion; *β*(*z*) is the thermal coefficient of the SOS; and *c*_0_(*z*) is the depth-dependent initial SOS. (Eq 1) can be rewritten as

$$\delta T(z)=\frac{c_{0}(z)}{2}k(z)\frac{\partial }{\partial z}\left(\delta t(z)\right),$$ (2)

where the coefficient $k\left(z\right)=\frac{1}{\alpha \left(z\right)-\beta \left(z\right)}$ is a medium-dependent parameter used to describe the confounding effects of changes in the SOS and thermal expansion when the temperature is increased. (Eq 2) can be simplified by assuming that *k* is a constant when the tissue temperature is less than 50°C [26–27]:

$$T(z)=\frac{c_{0}}{2}k\frac{\partial }{\partial z}\left(\delta t(z)\right)+T_{0}.$$ (3)

If the temperature at the tip of the RF electrode is known, the coefficient *k* corresponding to the ablation center in the tissue at each time point can be estimated using the echo time shift and the tissue temperature, as given by

$$k(z)=\frac{2\delta T(z)}{c_{0}}/\frac{\partial }{\partial z}\left(\delta t(z)\right).$$ (4)

However, using the single value of the coefficient *k* at the electrode tip cannot satisfy the calculation of ultrasound temperature imaging. Instead, a spatial distribution of local coefficients *k* (i.e., a two-dimensional *k* map) at each time point is required. For this reason, the adaptive-*k*-based ultrasound temperature estimation algorithm is proposed and the details are described below.

(i) At first, the initial *k* coefficient of the tissue was determined. Prior to RFA, the raw image data $r(x,y){|}_{t}{}_{=t_{0}}$ at the initial temperature *T*_0_ are collected. Assuming that the tissue is a homogeneous medium at thermal equilibrium, the *k*-value map $k(x,y){|}_{t=t_{0}}$ is a two-dimensional (2D) data matrix (the value of each pixel is the initial *k* coefficient) and the temperature image $T(x,y){|}_{t}{}_{=t_{0}}$ is a 2D map with pixel values corresponding to *T*_0_.
(ii) During RFA at the time point *t* = *t*_1_, the cumulative echo time shift map $\delta t\left(x,y\right){|}_{t}{}_{=t_{1}}$ is obtained from one-dimensional (1D) cross-correlation between $r(x,y){|}_{t}{}_{=t_{1}}$ and $r(x,y){|}_{t}{}_{=t_{0}}$. Assuming that there is no significant difference between the coefficients *k* at two adjacent time points, based on (Eq 3), the *k*-value map $k(x,y){|}_{t=t_{0}}$ is multiplied by differentiating $\delta t\left(x,y\right){|}_{t}{}_{=t_{1}}$ along the axial direction to obtain the temperature image $T(x,y){|}_{t}{}_{=t_{1}}$. Concurrently, $k(x,y){|}_{t}{}_{=t_{1}}$ is obtained using $\delta t\left(x,y\right){|}_{t}{}_{=t_{1}}$ and $T(x,y){|}_{t}{}_{=t_{1}}$ in (Eq 4).
(iii) Then, at time point *t* = *t*_2_, step (ii) is conducted to obtain $\delta t\left(x,y\right){|}_{t}{}_{=t_{2}}$, and $T(x,y){|}_{t}{}_{=t_{2}}$ is obtained using $\delta t\left(x,y\right){|}_{t}{}_{=t_{2}}$ and $k(x,y){|}_{t}{}_{=t_{1}}$ in accordance with (Eq 3). The *k*-value map $k(x,y){|}_{t}{}_{=t_{2}}$ is then obtained using $\delta \;t(x,y){|}_{t=t_{2}}$ and $T(x,y){|}_{t=t_{2}}$ by using (Eq 4).
(iv) Step (iii) is repeated to obtain the temperature images at each time point by
  $$T(x,y){|}_{t}{}_{=t_{i}}=\left(\frac{c_{0}}{2}\right)\left(k(x,y){|}_{t}{}_{=t_{i}-1}\right)\left(\frac{\partial }{\partial y}\delta \;t(x,y){|}_{t}{}_{=t_{i}}\right)+T(x,y){|}_{t}{}_{=t_{0}}$$ (5)

### Experimental setup and procedures

**Fig 1:**
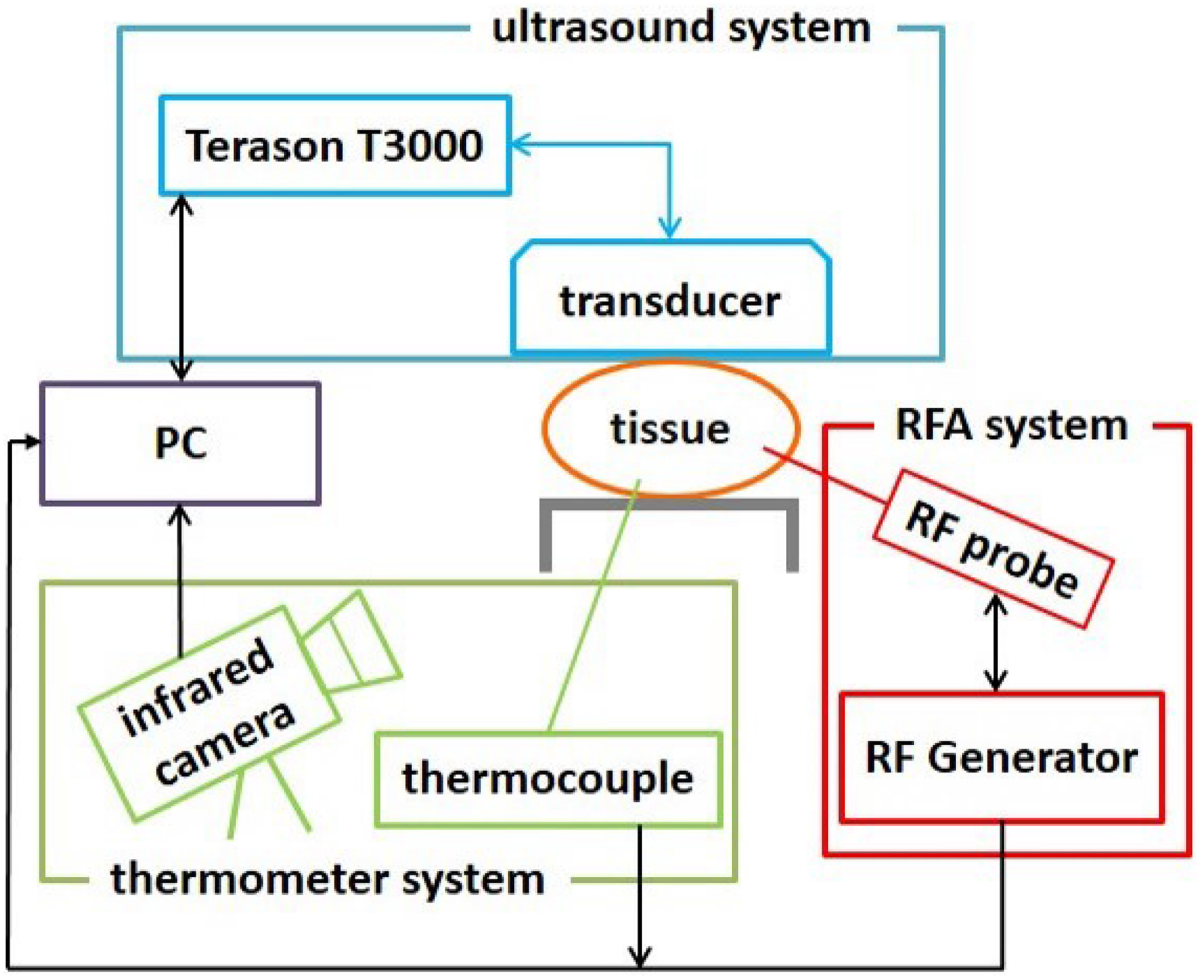
Illustration of the experimental setup. Ultrasound system was used to acquire backscattered signals from tissues for temperature imaging during RFA. The infrared camera was used to observe the 2D surface temperature distribution of the tissue sample, and thermocouples were inserted into the tissue to measure the internal ablation temperatures.

Fig 1 illustrates the experimental setup, which consisted of three systems: an ultrasound system (Model 3000, Terason, Burlington, MA, USA), a RFA system (Model VIVA RF generator, Starmed Co. Ltd., Goyang, Gyeonggi, South Korea), and a thermometer system comprising an infrared camera (DL770A, SUNRITE, Technology, Taipei, Taiwan) and thermocouples (TES-1384, TES Electrical Electronic Corp, Taipei, Taiwan). The ultrasound system equipped with a 7.5-MHz linear array transducer (Model 10L5, Terason) was used to acquire raw image backscattered data during RFA. The RFA system comprising a cool-tip RF electrode of length 1.5 cm (Model 17-20V15-40, Starmed Co. Ltd), an RF generator, a peristaltic pump, cables, and other accessories was used to ablate the tissues. The RF electrode had a built-in thermocouple that provided real-time feedback for the temperature at the tip of the electrode. The infrared camera was used to observe the surface temperature distribution of the tissue sample, and additional two thermocouples were inserted into the tissue to measure the temperatures at the ablation center and the lateral position (5 mm away from the electrode).

**Fig 2:**
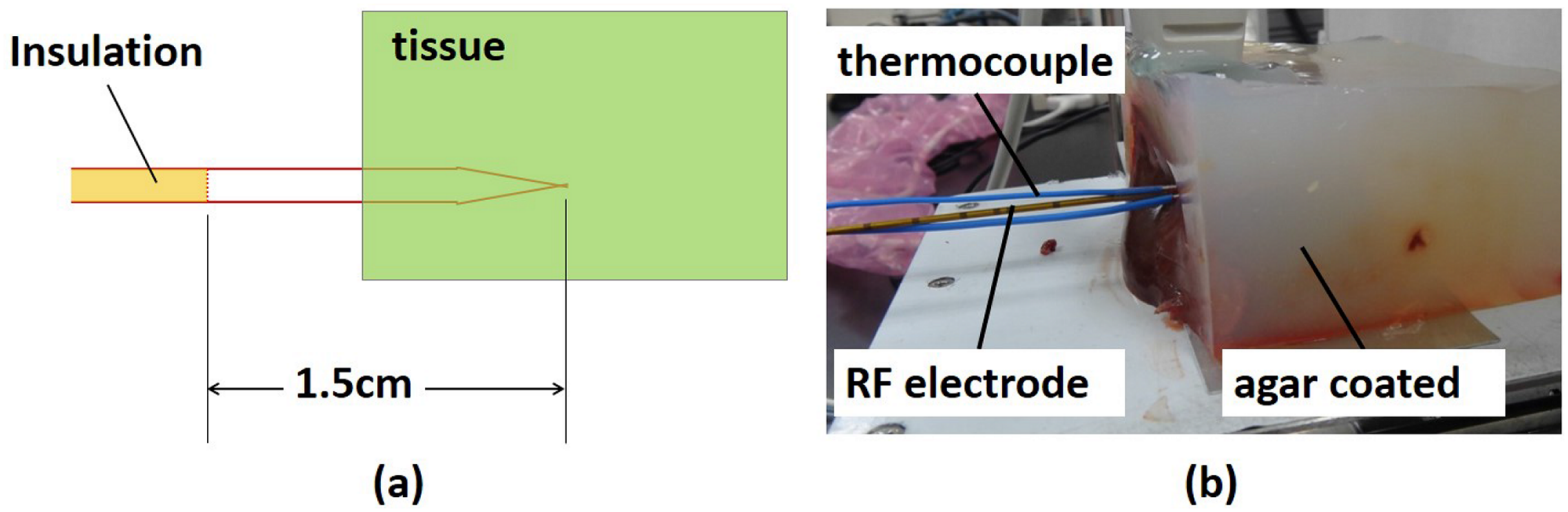
In the experimental design, only half of the RF electrode was inserted into the tissue, making heat transfer along with the cross-section of the tissue for monitoring temperature distribution by infrared imaging. (a) schematic diagram; (b) practical measurement arrangement.

In this study, porcine livers were used for *in vitro* validations of the proposed method. The coefficient *k* depends on tissue type and fat content [27]. According to the previous study, the coefficient *k* of porcine livers is 207.67°C [28], which was used as the initial value in the proposed algorithm. Subsequently, three groups of experiments were performed using RFA powers of 10, 15, and 20 W, respectively. In each group, five porcine livers obtained from local markets were used as *in vitro* tissue samples (total *n* = 15). Each liver sample was embedded in an agar phantom (Fig 2). One side of the phantom faced the infrared camera so that the infrared radiation emitted from the cross-section of the liver sample was directly detected by the infrared camera. In this arrangement, only half of the RF electrode (corresponding to a length of 8 mm) was inserted into the tissue, allowing the heat induced by the RF electrode to distribute in the cross-section of the tissue. Recall that the emissivity is 1 for a black body and is smaller than 1 for a gray body. A liver sample may be similar to a black body radiator because its emissivity is larger than 0.9 [23]. According to the calibration report provided by the manufacturer, the accuracy of the infrared camera is ±2°C.

During RFA with different powers, the infrared imaging system was used to monitor the surface temperature map corresponding to the cross-section of the tissue. The ultrasound transducer was placed as close as possible to the cross-section of the tissue sample to acquire ultrasound backscattered data at a sampling rate of 30 MHz. Ultrasound B-mode images were constructed using the absolute value of Hilbert transform of backscattered signals at a dynamic range of 40 dB. Temperature imaging was made using the proposed algorithmic procedure. The time intervals between each data acquisition were 0.5 and 5 seconds for ultrasound imaging and temperature measurements (infrared images and thermocouples), respectively. The gate length and overlap ratio used for 1D cross-correlation were 0.5 mm and 50%, respectively. An eighth-order low-pass Butterworth filter with a normalized cutoff frequency of 0.33 was used to reduce the number of ripples in the temperature estimates [14]. Finally, the temperature images obtained without and with the use of the adaptive-*k* method were compared with the infrared images. The temperatures as a function of time obtained from ultrasound temperature images, infrared images, and thermocouples were also compared for estimation error analysis.

## Results

**Fig 3:**
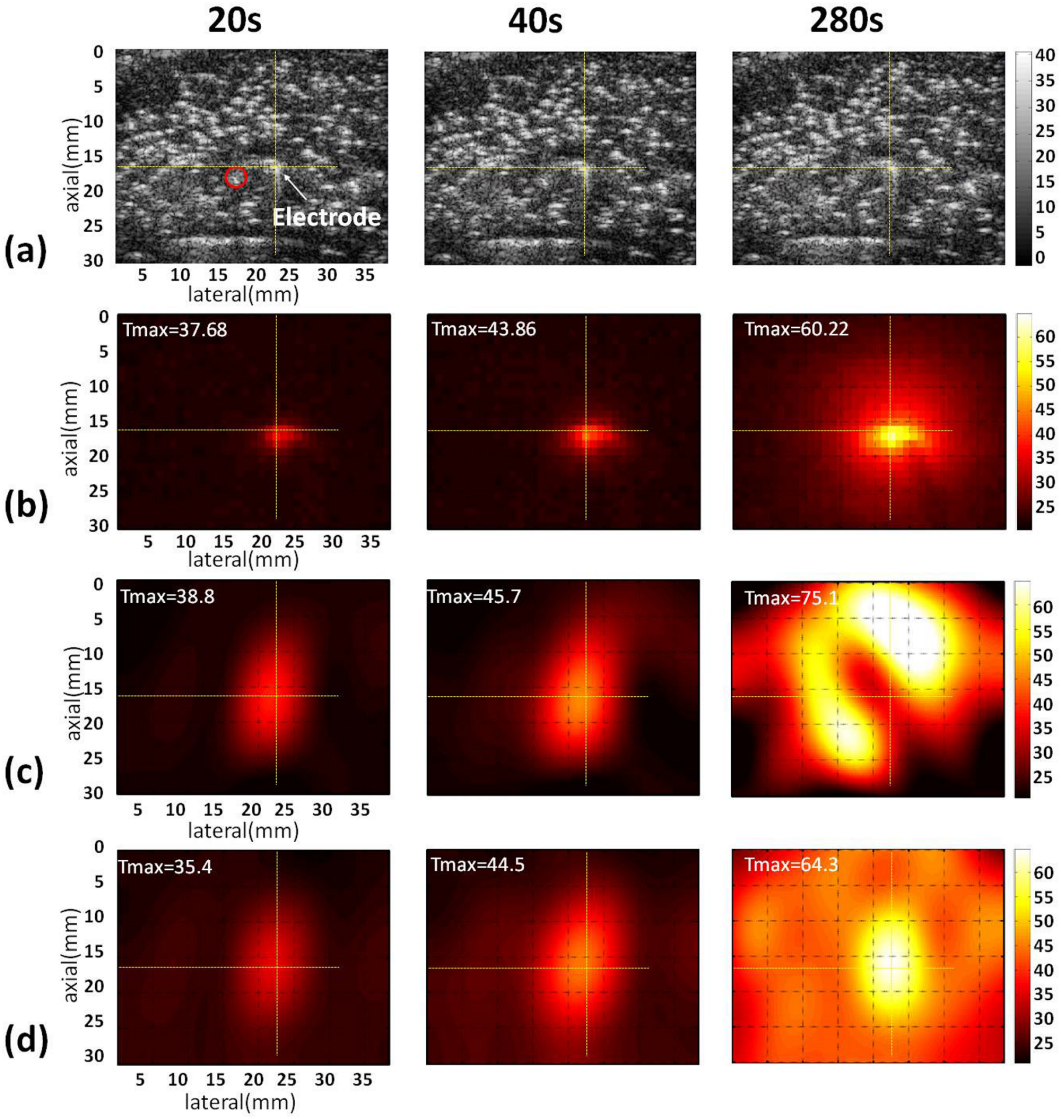
Typical images of the liver sample acquired at different time points during RFA of 10 W. (a) B-mode images; (b) infrared images; (c) temperature images constructed using constant *k*; (d) temperature images estimated using adaptive *k*. The yellow cross marks and red circles in the B-mode images indicate the locations of the RF electrode and thermocouples, respectively. The symbol “Tmax” is the maximum temperature in the temperature images.

**Fig 4:**
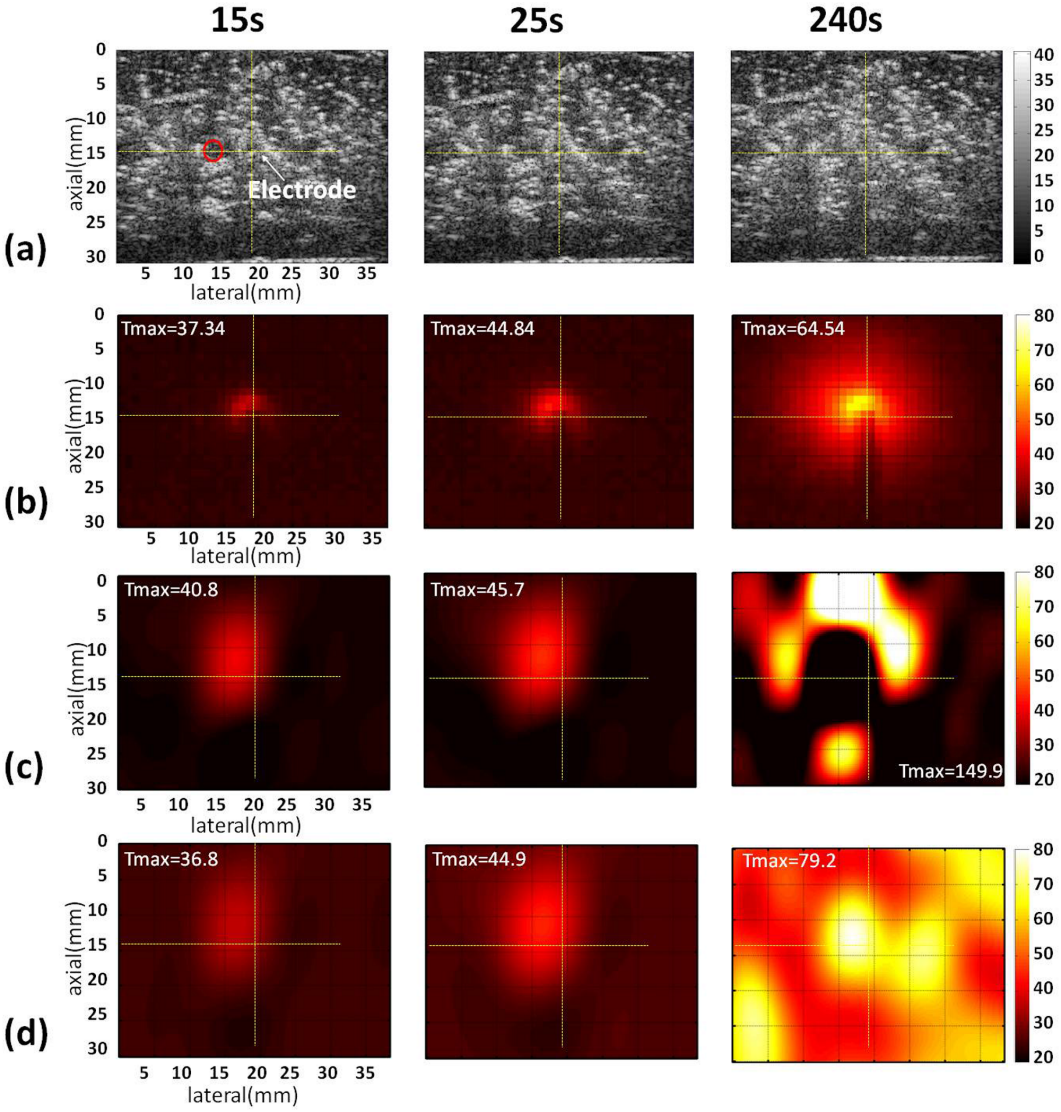
Typical images of the liver sample acquired at different time points during RFA of 15 W. (a) B-mode images; (b) infrared images; (c) temperature images constructed using constant *k*; (d) temperature images estimated using adaptive *k*. The yellow cross marks and red circles in the B-mode images indicate the locations of the RF electrode and thermocouples, respectively. The symbol “Tmax” is the maximum temperature in the temperature images.

**Fig 5:**
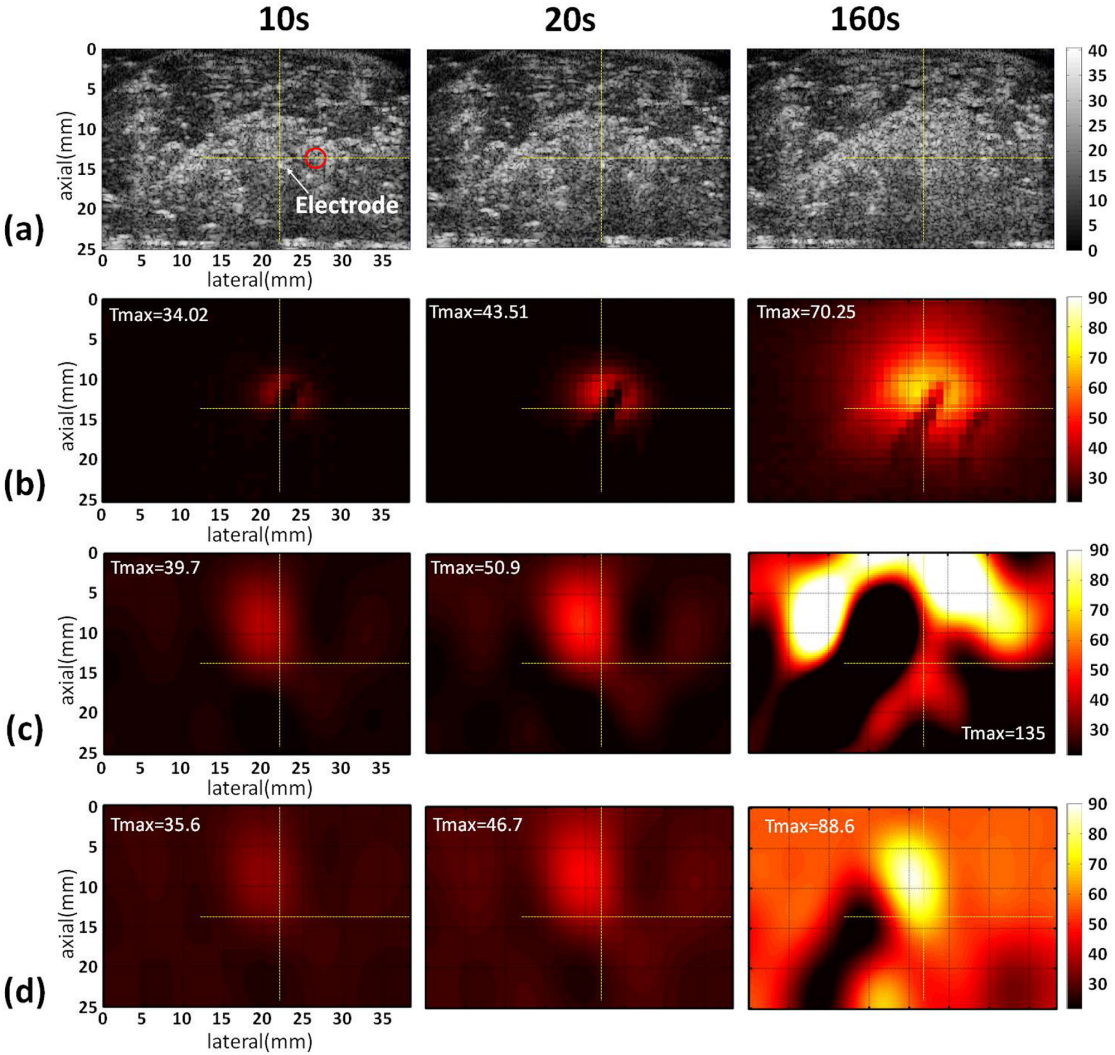
Typical images of the liver sample acquired at different time points during RFA of 20 W. (a) B-mode images; (b) infrared images; (c) temperature images constructed using constant *k*; (d) temperature images estimated using adaptive *k*. The yellow cross marks and red circles in the B-mode images indicate the locations of the RF electrode and thermocouples, respectively. The symbol “Tmax” is the maximum temperature in the temperature images.

Figs 3–5 show the B-mode, infrared, and temperature images of the liver sample obtained without and with the use of the adaptive-*k* method when different RFA powers (10, 15, and 20 W) were used. The yellow cross marks and red circles in the B-mode images indicate the locations of the RF electrode and thermocouples, respectively. Note that the RFA system automatically pauses when the tissue impedance is high enough to result in open circuit between the tissue and the ground. Typically, higher powers shorten the duration of RFA to have earlier end time points. At a power of 10 W, the conventional temperature images, obtained using constant-*k*, have distorted patterns and are inconsistent with the infrared images. By comparison, the proposed adaptive-*k* temperature imaging at different time points resulted in improved visualization of the temperature distribution (Fig 3). The superiority of adaptive-*k* temperature imaging over the constant-*k* method in describing the temperature distribution also can be found at powers of 15 and 20 W, although artifacts and location shift of temperature map did appear (Figs 4 and 5).

**Fig 6:**
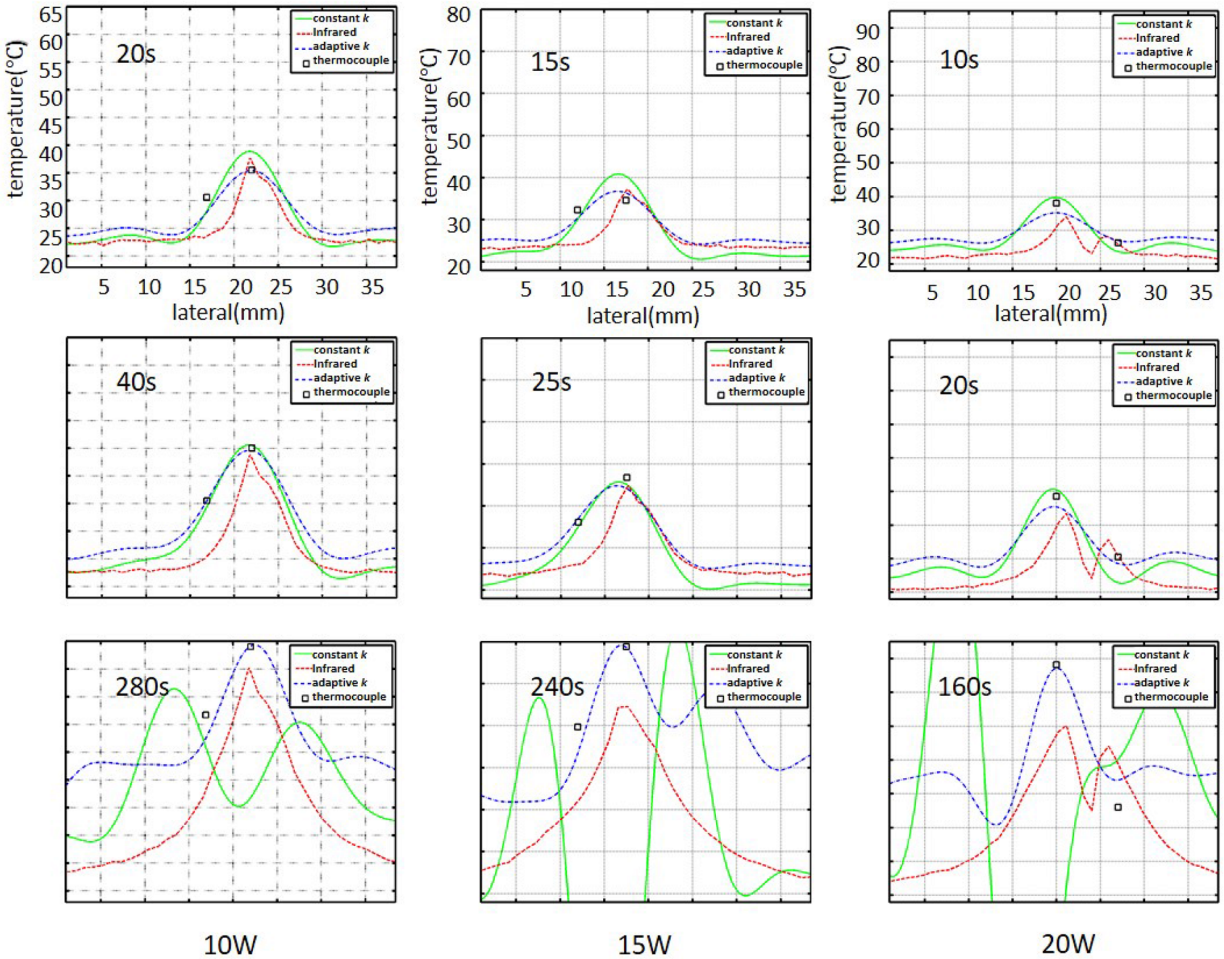
Comparisons of the lateral temperature profiles obtained using thermocouples, infrared, and ultrasound temperature imaging based on the constant- and adaptive-*k* methods. Black squares represent temperatures measured by thermocouples. Red lines represent the lateral profiles in the infrared image; green solid and dotted blue lines mean the lateral profiles of temperature imaging using constant *k* and adaptive *k*, respectively.

**Fig 7:**
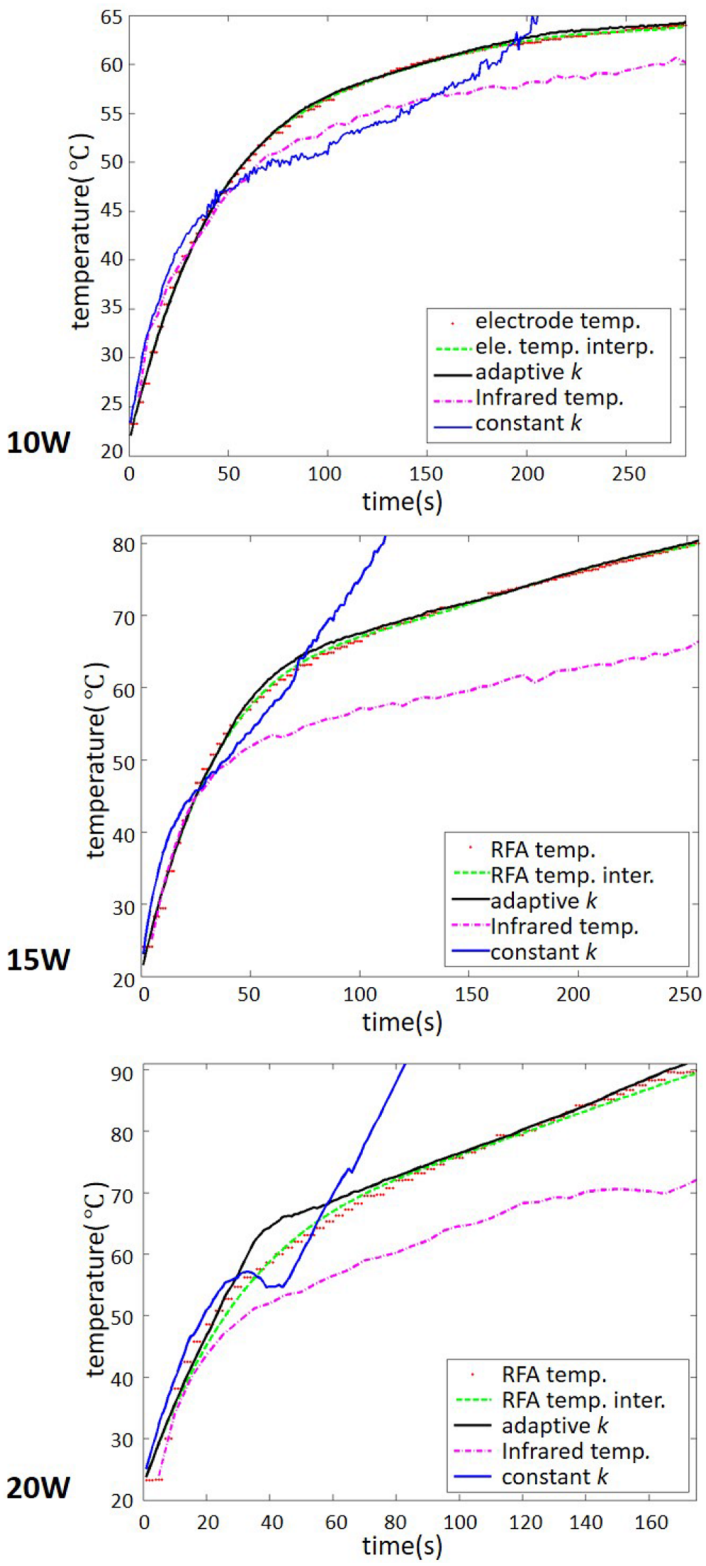
Temperatures measured by thermocouples and ultrasound temperature imaging as a function of ablation time during RFA of different powers. Red dotted curves represent the temperature of the RF electrode (the ablation center) measured by the thermocouple. Green dotted curves show the fitting curves of the red dotted data. Blue and black lines mean the temperature estimated using constant- and adaptive-*k*. Purple dotted curves represent the temperature changes in the infrared images.

**Fig 8:**
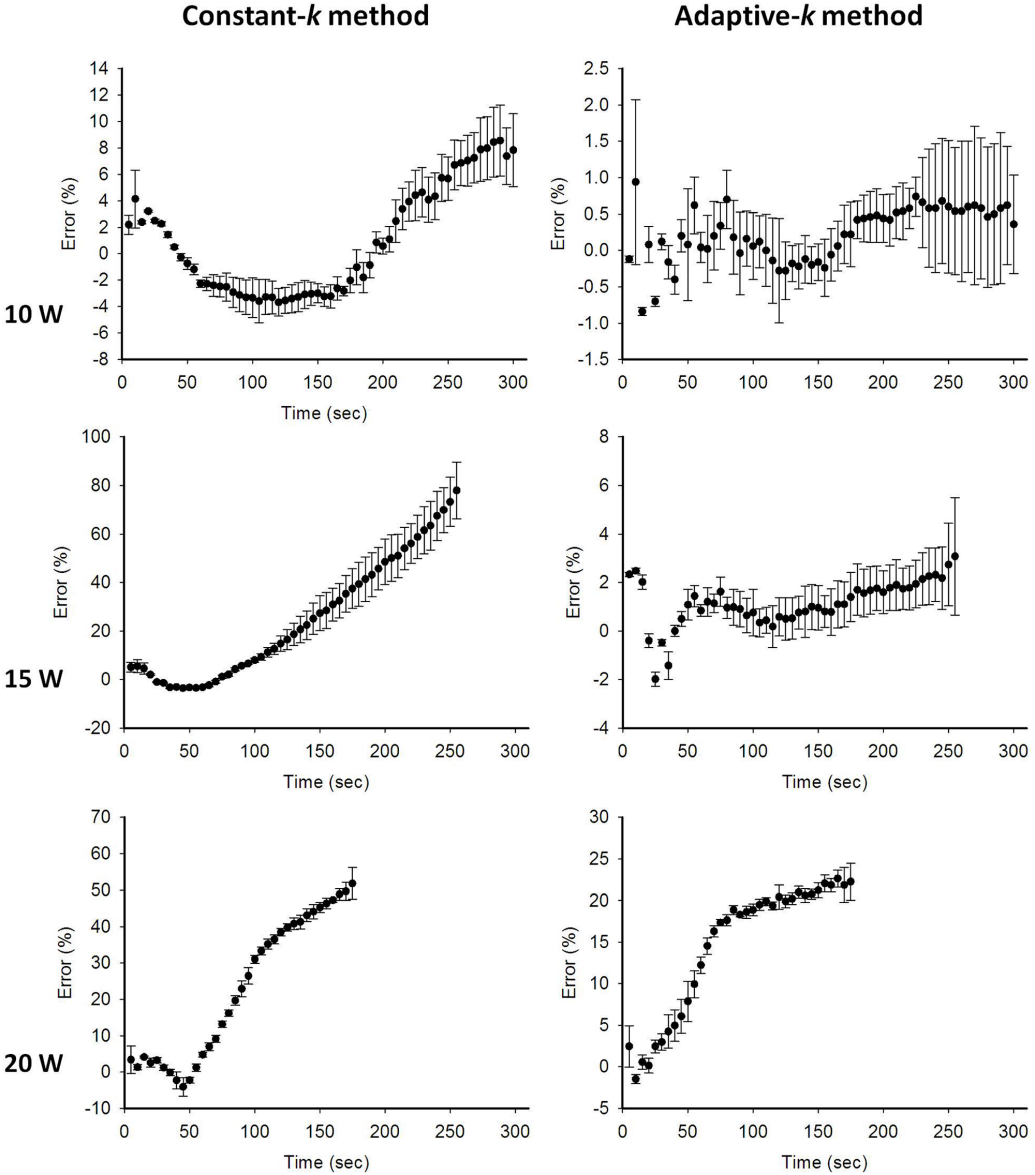
The estimation errors obtained using the constant- and adaptive-*k* methods in ultrasound temperature imaging of RFA at different powers. Compared with the conventional method using constant *k*, the proposed adaptive-*k* temperature imaging reduced the estimation errors to improve the accuracy of temperature estimate. Note that RFA system paused when the tissue impedance is high enough to result in open circuit between the tissue and the ground. Typically, higher powers shorten the ablation duration of RFA.

To confirm the above observations, the lateral temperature profiles obtained from the infrared images (red dashed lines) and temperature images (green lines: constant-*k*; dotted blue lines: adaptive-*k*) at different RFA powers were compared in Fig 6. The black squares represent the temperatures measured by the thermocouples inserted into the liver sample. The constant-*k* method was found to be inaccurate at describing the profiles of temperatures higher than 50°C. However, the lateral temperature profiles obtained using adaptive-*k* temperature imaging agreed favorably with those obtained using infrared imaging; in particular, the corresponding temperature values were close to those measured by the thermocouples even at temperatures higher than 50°C, as shown in Fig 7. It should be noted that the temperature values obtained using infrared imaging were lower than those measured using the thermocouple. This is because infrared imaging just shows the surface temperature of tissue samples for validations of temperature distribution and profile in adaptive-*k* temperature imaging. In order to further evaluate the estimation errors of the constant- and adaptive-*k* methods in temperature imaging, the temperature values measured from the thermocouple were used as the ground truth for comparisons, as shown in Fig 8. Under using the power of 10 W, compared with the estimation errors of the constant-*k* method varying between 2% and 10%, those of the adaptive-*k* method were approximately 2% during RFA. At the powers of 15 and 20 W, conventional temperature imaging produced errors larger than 50%, whereas adaptive-*k* temperature imaging reduced the estimation errors to smaller than 6% and 25%, respectively. The proposed adaptive-*k* method is capable of providing more precise temperature imaging at higher temperatures than constant-*k* ultrasound temperature estimation method.

## Discussion

This study proposed an adaptive-*k* algorithm for ultrasound temperature imaging based on echo time shift detection and explored its feasibility in monitoring changes in the temperature in tissues during RFA. The conventional algorithm, which uses a constant *k* coefficient, provided satisfying temperature estimation at temperatures lower than 50°C [23]. The limitation of the conventional temperature image constructed using the constant *k* has been discussed previously [14, 23, 29–30]. Compared with thermal expansion, changes in SOS dominate the variation in the echo arrival time of the RF signals acquired during the ablation process [31]. However, thermal expansion effects still contribute to errors in temperature maps. At temperatures higher than 50°C, thermal expansion can contribute to physical displacements to the same degree as apparent displacements contributed by SOS variations [32]. The present study demonstrated that using a dynamic *k* coefficient is an effective strategy for reducing measurement errors during temperature imaging, and this strategy provides estimates that are comparable to those measurements obtained using infrared imaging and embedded thermocouples. In particular, the proposed adaptive-*k* method was found to be feasible for a wide range of temperatures, including those higher than 50°C. Thus, it is more applicable for monitoring RFA than constant-*k* methods. Note that feedback [14] and model-based [24] adaptive methods have been used in echo-shift ultrasound temperature estimation for focused ultrasound hyperthermia with a temperature rise of no more than 10°C. To the best of our knowledge, this study is the first to introduce the adaptive-*k* echo-shift ultrasound temperature imaging for monitoring high-temperature RFA.

Although the proposed adaptive-*k* algorithm reduces the measurement errors of ultrasound temperature imaging in monitoring RFA, some problems are observed and need to be overcome further. As shown in Fig 3, the locations of the maximum value in adaptive-*k* temperature images well correspond to that of the RF electrode when the RFA power was 10 W. With increasing the RFA power, the location of the temperature peak in adaptive-*k* temperature images was gradually far away from that of the RF electrode (Figs 4 and 5). The above phenomenon was also found in the conventional temperature image using the constant *k*. A specific mechanism used to explain the location bias of heating center in temperature imaging is hard to conclude. However, we believe that two effects that degrade ultrasound temperature estimation methods may play roles to some degree. First, high powers provide near-boiling-point ablation to destroy tissues, and thus gas bubbles are induced to act as strong acoustic scatterers that alter the waveforms of backscattered signals [33] and increase the backscattered energy to alter local signal patterns or features [34]. Second, thermal expansion and irreversible tissue necrosis generated under high temperature changes SOS and tissue geometry, resulting in some artifacts in the temperature maps [35]. As shown in the B-mode images in Figs 4 and 5, tissues samples under RFA appeared to have image brightness enhancement and distortion in shape.

In the future, the proposed adaptive-*k* ultrasound temperature imaging can be combined with clinical ultrasound systems to evaluate the real-time thermal dosage during RFA. The proposed adaptive-*k* echo time shift detection algorithm is totally compatible with pulse-echo ultrasound systems because it utilizes RF signals for analysis. A previous study developed a real-time ultrasound temperature imaging system based on a commercial scanner and revealed that the computational kernel for real-time signal processing relies on the use of a multicore graphics processing unit [29]. While developing our real-time temperature imaging system, we also noted that a major obstacle to *in vivo* ultrasound temperature estimation is the effect of tissue motion. Some strategies have been previously proposed to address this issue [32,36]. Respiratory motion effects may be minimized by using high frame rate imaging or obtaining a temperature image within 2 to 3 seconds while subjects hold their breath. Using electrocardiography signals as the trigger to collect images or employing motion compensation using spatial interpolation and linear least-square fitting are also useful for reducing motion artifacts during temperature imaging. However, the existing methods of motion compensation still have limitations under more complex conditions, such as the involvement of rotation or other forms of deformation (e.g., lateral compression, warping) [37]. Implementation of real-time temperature imaging is challenging but is worthy of further investigation for future clinical applications.

## Conclusion

This study proposed an adaptive approach based on echo time shift detection that enabled the automatic adjustment of the coefficient *k* during ultrasound temperature monitoring of RFA. The results demonstrated that the proposed adaptive-*k* method improved the performance in visualizing temperature distributions, reducing the errors of temperature estimate when the ablation temperature of the tissue is higher than 50°C. The proposed strategy has potential to serve as a thermal dosage evaluation tool for monitoring high-temperature RFA.

## Supporting information

S1 Data Data of the estimation errors obtained using the constant- and adaptive-*k* methods in temperature imaging. (RAR)
